# Enteric neuromodulators and mucus discharge in a fish infected with the intestinal helminth *Pomphorhynchus laevis*

**DOI:** 10.1186/s13071-015-0970-7

**Published:** 2015-07-08

**Authors:** Giampaolo Bosi, Andrew Paul Shinn, Luisa Giari, Bahram Sayyaf Dezfuli

**Affiliations:** Department of Veterinary Sciences and Technologies for Food Safety, Università degli Studi di Milano, St. Trentacoste 2, 20134 Milan, Italy; Fish Vet Group Asia Limited, 99/386, Chaengwattana Building, Chaengwattana Rd., Kwaeng Toongsonghong, Khet Laksi, Bangkok 10210 Thailand; Department of Life Sciences & Biotechnology, University of Ferrara, St. Borsari 46, 44121 Ferrara, Italy

**Keywords:** Mucous cells, Enteroendocrine cells, Immunohistochemistry, Transmission electron microscopy, Confocal laser scanning microscopy, Acanthocephalan, *Pomphorhynchus laevis*, Host immunity, Infected intestine

## Abstract

**Background:**

In vertebrates, the presence of enteric worms can induce structural changes to the alimentary canal impacting on the neuroendocrine system, altering the proper functioning of the gastrointestinal tract and affecting the occurrence and relative density of endocrine cells (ECs). This account represents the first immunohistochemistry and ultrastructure-based study which documents the intimate relationship between the intestinal mucous cells and ECs in a fish-helminth system, investigating the potential effects of enteric neuromodulators on gut mucus secretion/discharge.

**Methods:**

A modified dual immunohisto- and histochemical staining technique was applied on intestinal sections from both infected and uninfected fish. Sections were incubated in antisera to a range of neuromodulators (*i.e.* leu-enkephalin, met-enkephalin, galanin and serotonin) and the glycoconjugate histochemistry of the mucous cells was determined using a subsequent alcian blue – periodic acid Schiff staining step. Dual fluorescent staining on sections prepared for confocal laser scanning microscopy and transmission electron microscopy were also used to document the relationship between ECs and mucous cells.

**Results:**

From a total of 26 specimens of *Squalius cephalus* sampled from the River Paglia, 16 (*i.e.* 62 %) specimens were found to harbour an infection of the acanthocephalan *Pomphorhynchus laevis* (average intensity of infection 9.2 ± 0.8 parasites host^−1^, mean ± standard error). When acanthocephalans were present, the numbers of mucous cells (most notably those containing acidic or mixed glycoconjugates) and ECs secreting leu-enkephalin, met-enkephalin, galanin, serotonin were significantly higher than those seen on sections from uninfected fish. The relationship between met-enkephalin-like or serotonin-like ECs and lectin DBA positive mucous cells was demonstrated through a dual fluorescent staining. The presence of tight connections and desmosomes between mucous and ECs in transmission electron micrographs provides further evidence of this intimate relationship.

**Conclusions:**

The presence of *P. laevis* induces an increase in the number of enteric ECs that are immunoreactive to leu- and met-enkephalin, galanin, and serotonin anti-sera. The mucous cells hyperplasia and enhanced mucus secretion in the helminth-infected intestines could be elicited by the increase in the number of ECs which release these regulatory substances.

## Background

In vertebrates, the mucosal immune system includes a unique collection of adaptive and innate immune cells and molecules whose synchronised functioning protects the host from a vast array of invasive agents [1]. The mucosal surface of the gastrointestinal tract is a complex organised system that includes the epithelium, immune cells and the resident bacterial community [2]. In teleosts, the skin, gills, urogenital system and gut are the principal mucosal surfaces and represent the first line of defence [1]. Mucus is an essential component of mucosal innate immunity [1, 3] with intestinal mucous cells playing a key role in controlling the inflammatory response [4].

Hyperplasia of mucous cells, with a resultant increase in mucus secretion, has been described in several fish-helminth systems [5, 6]. Definitive histometric data on the increased density of piscine intestinal mucous cells and the qualitative changes in the glycoconjugates secreted in response to helminth infection has been studied in detail by the current authors [7, 8].

The neuroendocrine system of the vertebrate gut includes the enteric nervous and diffuse endocrine systems (DES), both of which play important roles in modulating and co-ordinating a plethora of intestinal processes including secretion, absorption, immunity and motility [9]. A component of the DES are the endocrine cells (ECs) of the gut, which represent a highly specialised sub-population of cells [10] and there are numerous literary accounts regarding aspects of their immunohistochemistry and ultrastructure within the digestive tracts of uninfected fish species [9, 11–14]. In vertebrates, the presence of enteric worms can induce structural changes to the alimentary canal impacting on the neuroendocrine system, altering the proper functioning of the gastrointestinal tract and affecting the occurrence and relative density of ECs belonging to the DES [15–17].

In the current investigation, the occurrence of intestinal mucous cells and ECs secreting the neuromodulators leu-enkephalin, met-enkephalin, galanin and serotonin that are involved in mucus secretion were compared in specimens of chub, *Squalius cephalus* (L.), that were naturally parasitised with the acanthocephalan *Pomphorhynchus laevis* with uninfected counterparts. This account represents the first immunohistochemistry and ultrastructure-based study which documents the intimate relationship between the intestinal mucous cells and ECs of fish infected with an enteric worm. As part of this investigation a modified dual immunohisto- and histochemical staining technique is used with the principal aim of providing useful information on the potential effects of enteric neuromodulators on gut mucus secretion/discharge in a fish-helminth system.

## Methods

### Specimen collection and preparation

Twenty-six specimens of *S. cephalus* (average 26.2 ± 0.6 cm in total length, mean ± standard error S.E., range 20-30 cm) were caught by electrofishing in the River Paglia, a tributary of the River Tiber in Central Italy. Fish were anaesthetised in 125 mg L^−1^ MS222 (Sandoz, Basel, Switzerland) until opercular movement ceased and then their spinal cords were severed. Immediately after euthanasia, a complete necropsy was performed on each fish with particular interest paid to the gills, gonads, liver, kidney, spleen and alimentary canal for the presence of helminths. Fresh impression smears were prepared from each and screened for protistans. Of the fish examined, only a single helminth species, *i.e. P. laevis,* was encountered. Samples (15 × 15 mm) of each organ were taken including the sites at which the parasite was found attached to the intestine. The tissues were fixed in 4 °C Bouin’s fluid for 8 h. Thereafter, the specimens were dehydrated through an alcohol series and then paraffin wax embedded using a Shandon Citadel 2000 tissue processor. The absence of parasites in uninfected fish, established during the necropsy, was confirmed by examination of histological sections.

### Dual histo- and immunohisto-chemical staining procedure

To investigate the morphology of the cellular components and their secretions at the site of parasite infection, a modification of the dual, immunohisto- and histochemical staining procedure, which was described for the first time by Gulubova *et al*. [18], was employed. Histological sections of the acanthocephalan-infected mid-gut were incubated with the following anti-sera: a 1:500 dilution of anti-galanin (code AB5909, Millipore, USA); a 1:200 dilution of anti-leu-enkephalin (code CA-08-235, Genosys Biotechnologies Inc.); a 1:250 dilution of anti-met-enkephalin (code AB1975 Millipore, USA); and, a 1:1000 dilution of anti-serotonin (code AB938, Chemicon International Inc., Temecula, CA, USA) (for details see citations [16, 17, 19]). Thereafter, the same sections were then stained with alcian blue 8GX pH 2.5 and periodic acid-Schiff (AB/PAS) [20], to determine the glycoconjugates histochemistry of the mucous cells. Using the AB/PAS stain, acidic glycoconjugates stain blue with AB, neutral glycoconjugates stain purple with PAS, while cells containing mixed glycoconjugates appear violet with the combined AB/PAS stain.

### Evaluation of mucous and endocrine cells and data analysis

All stained histology sections were examined on and photographed using a Nikon Eclipse 80i microscope (Nikon, Tokyo, Japan) and associated computerised image analysis software (Nis Elements AR 3.0, Nikon). For quantitation of the histochemical and immunohistochemical preparations, the endocrine cells that were immunoreactive to each primary antisera and the mucous cells that stained either blue, purple or violet with the AB/PAS stain were evaluated in four different microscopic areas on each histological section. A total of five sections were assessed from each host; a total of 10 uninfected *S. cephalus* and 10 hosts bearing similar parasite burdens (*i.e.* average intensity of infection 9.8 ± 0.4 acanthocephalans host^−1^, mean ± S.E.; range 8-11 *P. laevis* host^−1^) were evaluated. The mean number of endocrine and mucous cells per 100,000 μm^2^ of epithelium in sections taken from uninfected and *P. laevis*-infected *S. cephalus* were compared using Student’s t-test. The level of significance was set at *p* = 0.01.

### Immunofluorescence staining

To better understand the intimate relationship between endocrine and mucous cells, a dual immunofluorescence reaction on intestinal sections was conducted. Sections (6-7 μm-thick) of the mid-gut of *P. laevis*-parasitised *S. cephalus* were dewaxed, re-hydrated, and then rinsed in Tris-buffered saline (TBS: 0.05 M Tris-HCl, 0.15 M NaCl) containing 0.1 % Triton-X 100 (TBS-T). To inhibit non-specific reactions, the histology sections were treated with 1:20 normal goat serum in TBS for 60 min in a humid chamber; sections were then incubated with the primary anti-serum: 1:200 rabbit polyclonal anti-met-enkephalin in TBS for 24 h at room temperature (RT) or 1:200 rabbit polyclonal anti-serotonin in TBS for 24 h at RT. Slides were then washed in TBS-T before they were treated with avidin-biotin blocking solutions as per the manufacturer’s guidelines (Vector Lab., USA). Thereafter, the sections were rinsed in TBS-T and incubated with 10 μg ml^−1^ goat biotinylated anti-rabbit IgG (Vector Lab.) in TBS for 2 h at RT. The sections were then rinsed twice in TBS-T and then treated with 10 μg ml^−1^ fluorescein avidin D (Vector Lab.) in 0.1 M NaHCO_3_ pH 8.5 with 0.15 M NaCl for 2 h at RT.

The slides incubated for the demonstration of met-enkephalin-like or serotonin-like immunofluororeactivity were treated with 10 μg ml^−1^ biotinylated *Dolichos biflorus* agglutinin (DBA, Vector Lab.) in 10 mM HEPES pH 7.5, 0.15 M NaCl, 0.08 % sodium azide, 0.1 mM CaCl_2_ for 3 h at RT. DBA is a glycoprotein, isolated from the horse gram seed with a high binding affinity for N-acetylgalactosamine residues that are widely present in mucins and previous trials have demonstrated that DBA is a good marker for the intestinal mucous cells of fish [21, 22]. After the treatment, the sections were rinsed in TBS-T and then incubated with 10 μg ml^−1^ rhodamine avidin D (Vector Lab.) in 0.1 M NaHCO_3_ pH 8.5 with 0.15 M NaCl for 2 h at RT. The stained tissue sections were then mounted with Vectashield® mounting medium (Vector Lab.) and examined on a Zeiss 510 confocal laser scanning microscope (CLSM). The possible co-occurrence of met-enkephalin- and serotonin-like substances within the intestinal endocrine cells of acanthocephalan-infected fish, a double immunofluorescence reaction combining the two antisera on the same slide was performed. All sections were excited using multi-argon/helio-neon-green lasers with excitation and barrier filters set for fluorescein and rhodamine. Green and red fluorescent signals were obtained concurrently through alternate excitation (0.2 s^−1^) at 488 nm and 540 nm, respectively. Under these viewing conditions, there was no cross-contamination of the two signals.

Sections of mammalian (*i.e.* swine and rat) tissues were used as positive controls, whereas negative controls were obtained by the omission of the primary antisera or the lectin on representative sections from infected and uninfected hosts. Both sets of controls gave the expected results.

### Transmission electron microscopy

For electron microscopy, representative pieces (7 × 7 mm) of *P. laevis*-infected and uninfected intestines were fixed in 2.5 % glutaraldehyde in 0.1 M cacodylate buffer for 3 h at 4 °C before being post-fixed in 1 % osmium tetroxide in the same buffer for 3 h. The samples were then dehydrated through a graded acetone series before being embedded in epoxy resin (Durcupan ACM, Fluka). Semi-thin sections (*i.e.* 1.5 μm) were cut on a Reichert Om U 2 ultramicrotome using glass knives and then were stained with toluidine blue. Ultra-thin sections (*i.e.* 90 nm) were stained with a 4 % uranyl acetate solution in 50 % ethanol and Reynold’s lead citrate and then were examined using a Hitachi H-800 electron microscope.

## Results

From a total of 26 specimens of *S. cephalus* sampled from the River Paglia, 16 (*i.e.* 62 %) specimens were found to harbour an infection of the acanthocephalan *P. laevis* (3-15 parasites host^−1^, average intensity of infection 9.2 ± 0.8 parasites host^−1^, mean ± S.E.). Most acanthocephalans were found attached in the mid-gut region of their hosts as the favoured sites. No other protistan or helminth species were encountered.

### Histochemical and immunohistochemical staining of intestinal sections

**Fig. 1:**
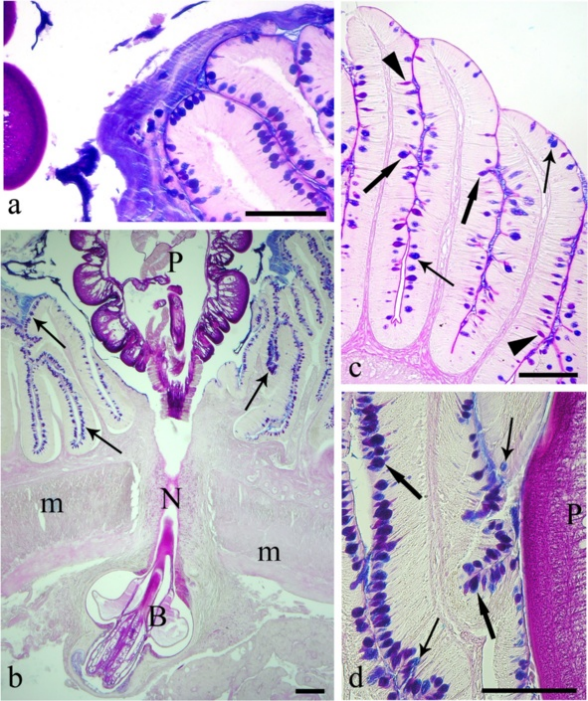
**a** The intestinal mucosa of the infected *Squalius cephalus* is covered by a thick blanket of mucus positive to alcian blue (AB). **b** Intestinal wall of a specimen of *S. cephalus* parasitised by the acanthocephalan *Pomphorhynchus laevis* which has penetrated through all the tissue layers of the intestine. The extroversible open bulb with hooks (B) is surrounded by fibrous inflammatory tissue, while the neck (N) at the point of attachment has disrupted the muscle layers of the host (m), and the body of parasite (P) impairs the intestinal folds, which show a high number of mucous cells in its epithelium (arrows). **c** Intestinal mucosa of an uninfected *S. cephalus* with a low number of mucous cells containing either acid (thin arrows), neutral (arrowheads) or mixed glycoconjugates (thick arrows). **d** High magnification of the intestinal epithelium in close proximity to the parasite (P) showing a high density of acid (AB-stain, thin arrows), and mixed (AB-periodic acid Schiff stain, thick arrows) glycoconjugates in the mucous cells. Scale bars 100 μm

**Fig. 2:**
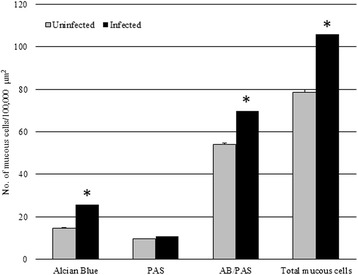
The mean number of mucous cells (per 100,000 μm^2^) in uninfected *Squalius cephalus* (grey columns) compared with conspecifics parasitised with the acanthocephalan *Pomphorhynchus laevis* (black columns). Mucous cells were counted for their staining affinity to alcian blue (AB), to periodic acid-Schiff (PAS) or to their combination (AB/PAS). Error bars represent the mean standard error, while the asterisk indicates a significant difference between groups (*p* < 0.01)

In the histological sections cut from the acanthocephalan-infected intestines, an excessive mucus, which appeared as a thick, adherent blanket that gave an intense positive signal when stained with alcian blue (Fig. 1a, b), was evident. The study found that when acanthocephalans were present, the total mucous cell number increased significantly (t = - 13.887, *p* = 0.000; Figs. 1a, b, d and 2). Likewise, the number of mucous cells containing either acidic glycoconjugates (*i.e.* staining blue) or mixed glycoconjugates (*i.e.* staining violet), also were found to have increased significantly (t = -11.631, *p* = 0.000, and t = -12.271, *p* = 0.000, respectively; Figs. 1a, b, d and 2).

In the sections cut from the uninfected intestinal tracts of *S. cephalus*, the mucous cell community consisted of predominantly mixed glycoconjugates (*i.e.* acidic and neutral), evident by their violet stain with AB/PAS (Figs. 1c and 2).

**Fig. 3:**
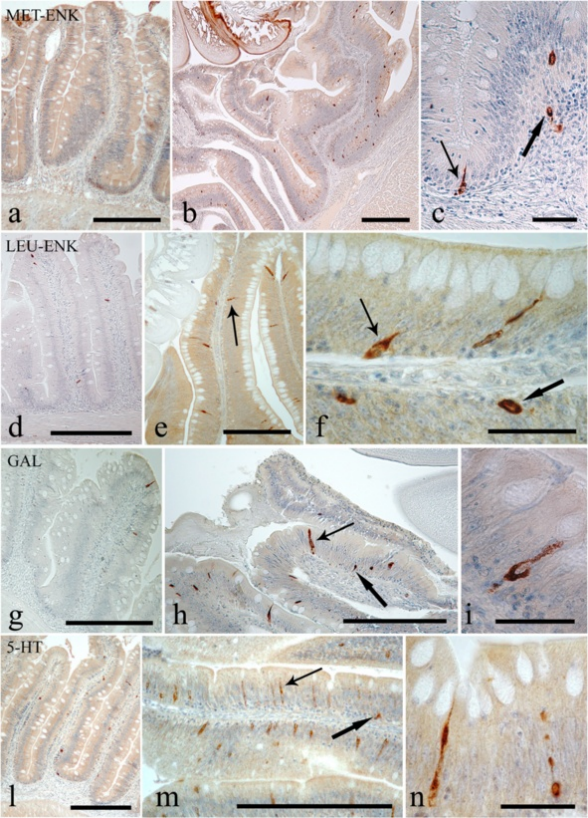
Immunoreactivity of the endocrine cells within the intestine of *Squalius cephalus* to met-enkephalin (**a**-**c**), leu-enkephalin (**d**-**f**), galanin (**g**-**i**), and to serotonin (**l**-**n**) antisera. The sections taken from the mid-guts of uninfected hosts (**a**, **d**, **g**, **l**) always have a lower number of immunopositive endocrine cells than those infected with the acanthocephalan *Pomphorhynchus laevis* (**b**, **e**, **h**, **m**). Both the open type (thin arrows) and closed type (thick arrows) immunoreactive endocrine cells can be seen (**c**, **f**), together with the open type immunoreactive endocrine cells that possess a mid-epithelial body (**i**), or with those that appear “reservoir-like” (n; see Results section). Scale bars: a, b, d, e, g, h, l, m: 100 μm; c, f, I, n: 20 μm

**Fig. 4:**
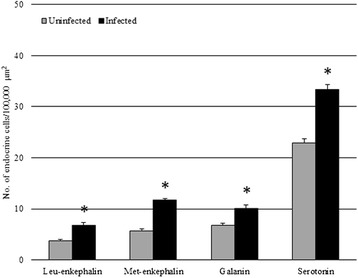
The mean number of enteroendocrine cells (per 100,000 μm^2^) immunoreactive to the specified antisera within the uninfected intestines of *Squalius cephalus* (grey columns) and in counterparts infected with the acanthocephalan *Pomphorhynchus laevis* (black columns). Bars represent the mean standard error while the asterisk indicates a significant difference between groups (*p* < 0.01)

**Fig. 5:**
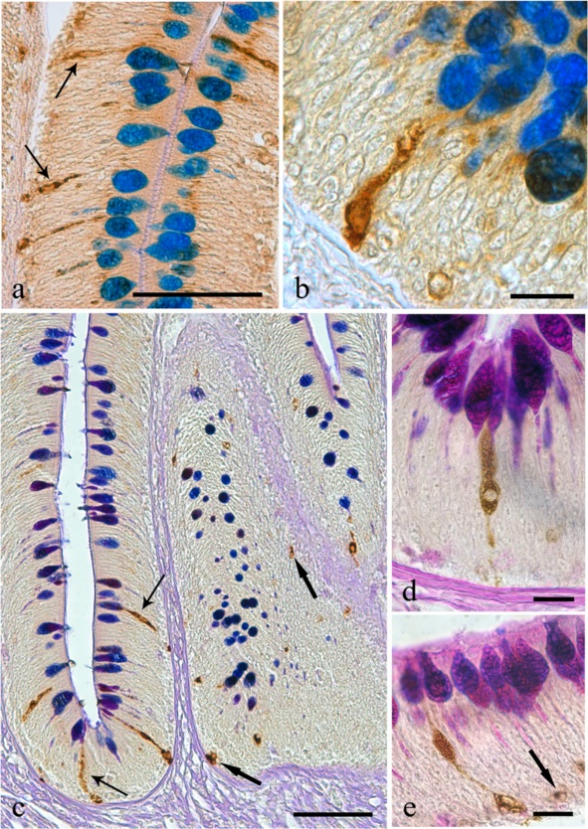
Histological sections from the intestinal mucosa of *Squalius cephalus* parasitised by the acanthocephalan *Pomphorhynchus laevis* and treated with either the leu-enkephalin (**a**-**b**) or met-enkephalin (**c**-**e**) antisera followed by a subsequent AB/PAS stain for mucous cells. Several immunoreactive endocrine epithelial cells can be seen of which many are in close contact with the goblets of the mucous cells. Immunoreactive endocrine cells with differing morphologies can be seen and include: those of the closed type (c, e, thick arrows), the typical open type (a, c, thin arrows), the open type with a mid-epithelial body (**d**), and, the open type with a “reservoir-like” appearance to them (**b**, **e**). Scale bars: a, c: 50 μm; b, d, e: 10 μm

**Fig. 6:**
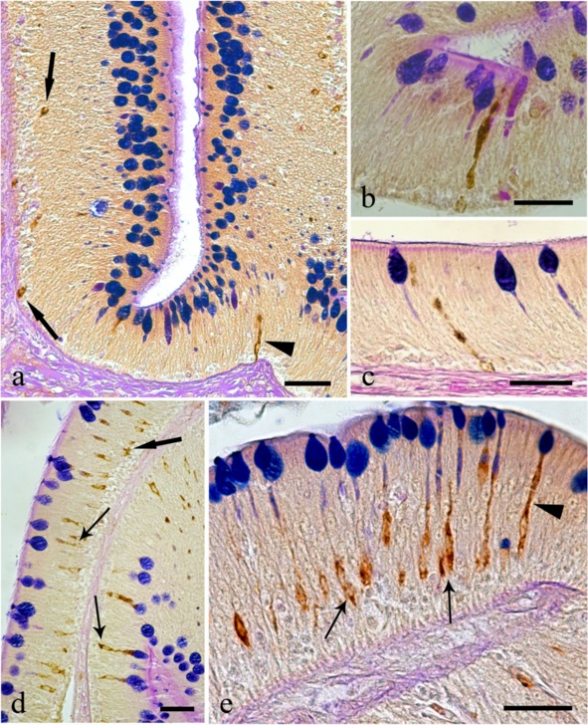
Histological sections from the intestines of *Squalius cephalus* infected with the acanthocephalan *Pomphorhynchus laevis* and treated with either the galanin (**a**-**c**) or the serotonin (**d**-**e**) antisera and a subsequent AB/PAS stain for mucous cells. There are numerous endocrine cells that are immunoreactive to the two antisera; many of these are close to the goblets of the mucous cells. The immunoreactive endocrine cells also display a range of differing morphologies including: the closed type (a, d, thick arrows), the open type with a mid-epithelial body (d-e, thin arrows), and, the open type with a “reservoir-like” appearance to them (a-c, e, arrowheads). Scale bars 20 μm

Endocrine cells that were immunoreactive (IR) to each of the four anti-sera were seen in the intestinal sections of *S. cephalus* infected with *P. laevis* (Figs. 3 and 4). The number of IR cells for each anti-sera in the sections taken from acanthocephalan-infected hosts were significantly higher than those seen on sections from uninfected hosts (Figs. 3 and 4). Most of the IR ECs that were seen were of the open type, while the closed types were only encountered occasionally (Fig. 3c, f). Among the open type of IR ECs, there were a group of cells that were characteristically sub-triangular (Fig. 3c, f), and a second group that typically possessed a thin base, a large medial portion which contains the nucleus (Fig. 3f, i) or very few protuberances along its apical process that runs towards the epithelial surface (Fig. 3n). This second group of ECs will in this account be subsequently referred to as “reservoir-like cells” because of their general resemblance to select waterbodies and to help distinguish these cells from the first group of sub-triangular shaped ECs. The dual staining protocol combining a immunohistochemistry step with AB/PAS staining helps demonstrate that there is a close relationship between the ECs that immunoreactive to anti-leu-, -met-enkephalin, -galanin and -serotonin sera and the apical, large mucin granules of the mucous cells (Figs. 5 and 6).

### Immunofluorescence

**Fig. 7:**
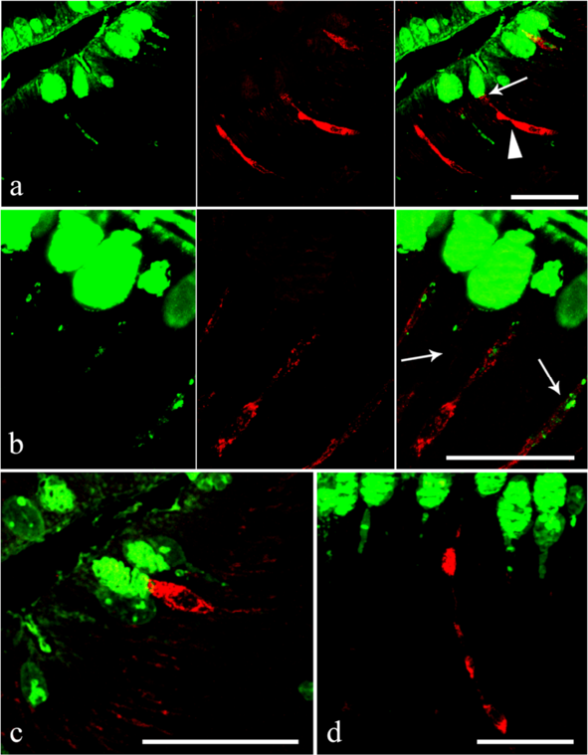
**a** Within the acanthocephalan infected intestines of *Squalius cephalus*, the goblets of the mucous cells in the intestinal epithelium are shown to be immunofluroreactive to DBA (left image), while the endocrine cells are seen to be immmunofluroreactive to the met-enkephalin antiserum (middle image). Note the “reservoir-like” shape of the endocrine cells (arrowhead) and the close contact between a mucous cell and an endocrine cell (thin arrow, right image). **b** Mucous cells that are immunofluororeactive to the DBA lectin (left image). The middle image shows three endocrine cells, in a section taken from the intestine of an infected host, that are immunofluororeactive to the serotonin antiserum. The cytoplasmic processes of the immunofluororeactive endocrine cells appear to envelope the cytoplasm of the mucous cells (thin arrow, right image). **c** The apical portion of an endocrine cell that possesses a mid-epithelial body that is immunofluororeactive to the met-enkephalin antiserum; this cell is close to the apical goblet of a mucous cell. **d** An endocrine cell with a “reservoir-like” appearance within the gut epithelium of an infected host that is immunofluoropositive to the serotonin antiserum. Scale bars 20 μm

As the “reservoir-like” ECs resemble differentiating mucous cells, to avoid an inexact identification a double immunofluorescence protocol was performed that uses the lectin DBA and the sera anti-met-enkephalin and/or anti-serotonin. DBA clearly binds to mucins in the mucous cells, whereas the met-enkephalin- or serotonin-like substances were observed in the ECs (Fig. 7). The apical cytoplasmic processes of the immunofluororeactive ECs were often seen in close proximity to mucous cells (Fig. 7a), in much the same manner as seen with the cells stained using the combined immunohistochemical and stained glycoconjugates protocol (Figs. 5 and 6).

**Fig. 8:**
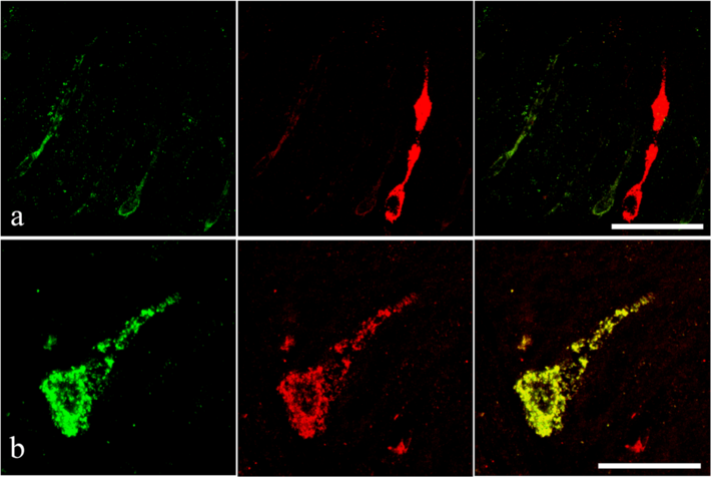
**a** Endocrine cells from the intestinal epithelium of an acanthocephalan infected specimen of *Squalius cephalus* that are immunofluororeactive to the serotonin antiserum (left image) and contain a met-enkephalin-like substance (middle image). Note the “reservoir-like” appearance of the met-enkephalin immunofluoropositive endocrine cell (middle and right images). **b** An open type endocrine cell that is immunofluororeactive to both the anti-met-enkephalin (left image) and to the anti-serotonin (middle image) anti-sera. The right image shows the co-localisation of the two neuromodulators within the same endocrine cell. Scale bars 20 μm

The double immunofluorescence reaction using the two antisera demonstrates that in the mid-gut of acanthocephalan-infected *S. cephalus,* there are ECs that contain either met-enkephalin- or serotonin-like substances (Fig. 8a), and that in certain occurrences there are ECs which contain both (Fig. 8b).

### Transmission electron microscopy

**Fig. 9:**
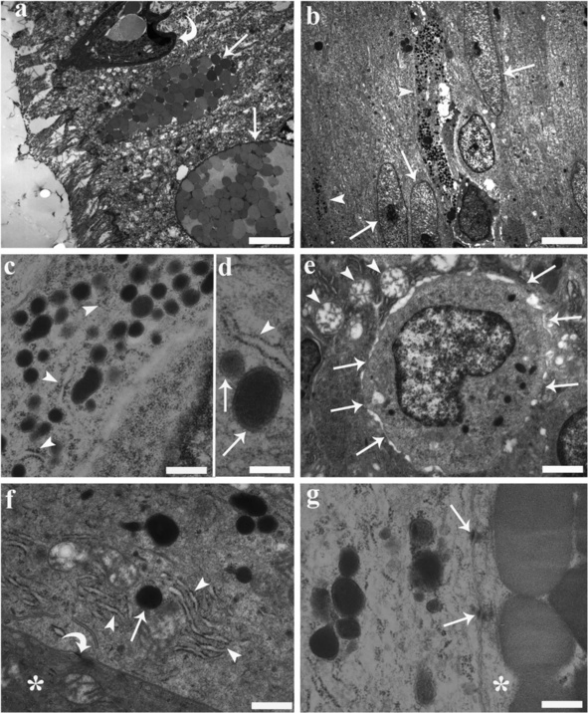
Transmission electron micrographs from the mid-gut of a specimen of *Squalius cephalus* infected with *Pomphorhynchus laevis.* **a** Two mucous cells (arrows) containing numerous mucus granules of various size and electron densities that are positioned close to the surface of the epithelium. The curved arrow highlights the presence of a rodlet cell. **b** Two endocrine cells (arrow heads) with long, narrow extensions directed toward the lumen are seen positioned between gut enterocytes (arrows). **c** Numerous secretory granules within the cytoplasm of an endocrine cell that is in close proximity to rough endoplasmic reticulum (RER) (arrow heads). **d** A fine electron-dense material can be seen filling the inner part of the granules (arrows); an electron-lucent halo separates the core of the granule from the membrane; the relationship between the RER (arrow head) and granules is visible. **e** The electron micrograph shows the basal portion of an endocrine cell that contains an euchromatinic nucleus and has numerous connections (arrows) with the surrounding mucous cells. Note the round mitochondria (arrow heads) with moderate cristae within the mucous cells. **f** An endocrine cell adjacent to a mucous cell (asterisk), with well-developed RER (arrow heads) close to a secretory granule (arrow). Also note the different electron-densities of the cytoplasm of the two cells and the presence of a desmosome (curved arrow). **g** An endocrine cell connected to a mucous cell (asterisk) by two desmosomes (arrows). Scale bars: a: 3.14 μm; b: 3.57 μm; c: 0.39 μm; d: 0.25 μm; e: 1.06 μm; f: 0.55 μm; g: 0.33 μm

Mucous cells were seen scattered among the enterocytes of the mid-gut, extending from the basal membrane toward the luminal side of the intestine. The mucous cells are readily distinguished by their large, oval euchromatinic nucleus and by their electron-dense cytoplasm, the supernuclear portion of which commonly contains numerous mucus granules of differing sizes and electron-densities (Fig. 9a). These cells had numerous, round mitochondria with a moderate number of cristae (Fig. 9e), a well-developed rough endoplasmic reticulum and numerous free ribosomes. The mucous cells were seen to make tight connections (Fig. 9e) and desmosomes (Fig. 9f, g) with the ECs.

Similarly, the ECs of the *S. cephalus* mid-gut were readily characterised by the shape and electron density of their secretory granules. The endocrine cells with a basal euchromatinic nucleus were approximately pyramidal (Fig. 9b) and were seen to extend from the basal membrane toward the gut lumen. Most of the ECs that were encountered were of the open type with a secretory granule-containing base and a long narrow extension directed toward the lumen (Fig. 9b). Some ECs though were seen lodged between the basal portion of some epithelial cells without a free border toward the gut lumen. The cytoplasm of the ECs were lighter than those of the surrounding cells (Fig. 9f) and were observed to contain numerous round to oval shaped secretory granules (Fig. 9c); the apical portion contained fewer granules. The secretory granules were of approximately similar sizes and were filled with a finely electron-dense material (Fig. 9c); in some granules, an electron-lucent halo separating the membrane from the core was visible (Fig. 9d). Free ribosomes and rough endoplasmic reticulum with long cisternae were frequently seen next to secretory granules (Fig. 9c, d, f). These endocrine cells contained few mitochondria and did not possess well-developed Golgi complexes. Several tight connections (Fig. 9e) in the basal part of the cell made with the mucous cell and other adjacent cells could be seen. The plasma membrane which covers the endocrine cell, in the middle portion of the cell appears to be closely connected to the neighbouring intestinal mucous cells and enterocytes by desmosomes (Fig. 9f, g).

## Discussion

The gastrointestinal tract is typically covered by mucus, the properties of which change in different regions of the alimentary canal [3]. The mucus, which can be considered an aggregate secretion, is produced by mucous cells; mucus, however, differs from mucin which refers to specific glycoproteins within this secretion [23]. Intestinal mucus, which is continuously secreted to renew the coating [24], is a dynamic system which is coupled to the immune system [25]. An accelerated secretion is characterised by a rapid, massive mucous cell discharge in response to physiologic or pathologic stimuli [26].

In the current study, a highly significant increase in the number of mucous cells was seen within the *P. laevis*-infected intestines of *S. cephalus* when compared to uninfected counterparts. The presence of an excessive mucus in the histology sections processed from all infected hosts was evident. A heavy mucus production has also been described from several other fish-helminth systems including those detailed by Chambers *et al*. [5], Alvarez-Pellitero [6], Bosi *et al*. [7], and, Dezfuli *et al*. [8]. The attachment organ of enteric helminths often provokes an inflammatory response within the host’s gastrointestinal tract [27]. Inflammation is a protective reaction in response to injury which results in an array of specific chemical and morphological alterations to the cellular community and tissues at the site of infection [28]. Intestinal mucous cells have a key role in controlling inflammation and there is now a general acceptance that these represent principal immune cells [4]. Through continued research into piscine mucosal immunity it is anticipated that in addition to gaining greater clarity on the immune defence mechanisms of fish, we will move to gain critical insights on some of the current unresolved paradigms in mammalian mucosal immunity [29].

Mucus secretion is regulated by a range of neural, hormonal, and paracrine signals [15, 26]; during inflammation, for example, a variety of peptides and inflammatory mediators are involved in regulating the release of mucus [26]. In vertebrates, gut ECs are recognised by the expression of an array of regulatory molecules, which modulate the functioning of the mucosal immune response [1, 9]. While the effects of ECs on intestinal mucus secretion and discharge in mammalian system have been comprehensively studied [26], there are only a few such studies that have been undertaken in fish [30]. Of the studies that have been conducted, the focus has been on immunohistochemistry of the regulatory substances the ECs produce [9, 14, 30–32] or on ultrastructural features of the cells [11–13, 33–35]. In the *S. cephalus*-*P. laevis* system considered here, there was a significant increment in the number of ECs containing leu-enkephalin, met-enkephalin, galanin and serotonin. An earlier study by Dezfuli *et al*. [19] also observed an increase in the number of ECs immunoreactive to met-enkephalin in the intestines of brown trout, *Salmo trutta* L., infected with cestodes, while a study conducted by Bermúdez *et al.* [32] found an increase in the number of ECs immunoreactive to serotonin in turbot, *Scophthalmus maximus* (L.), infected with myxozoans.

Leu- and met-enkephalin are opioid peptides that are commonly found within the gut neuroendocrine system of teleosts [36]. Generally, opioids are thought to regulate the activity of intestinal motility [37], and to modulate the innate and acquired immune systems [38]. Opioid peptides play an important role in the discharge mechanism of mucus from goblet cells, induced by luminal stimuli [39]. Within the fish’s intestine, galanin acts as a cholinergic co-mediator [40, 41], whereas the release of mucus from goblet cells is stimulated by cholinergic agonists [42]. Serotonin is involved in mucus secretion [26] and in the regulation of epithelium [13]. Serotonin has been shown to induce the release of mucin in isolated loops of rat small intestine [43], and to result in a marked rise in colonic mucin secretion in the perfused colon of rats [26]. In the intestine of acanthocephalan-infected *S. cephalus*, a significant increase in the number of anti-leu- and met-enkephalin, galanin, and serotonin IR ECs was seen suggesting that these four neuromodulators may be involved in the hyperplasia of mucous cells and in enhanced mucus discharge.

Within the intestines of mammals, mucus secretion may be induced by several factors including the classical neurotransmitters, gut neuropeptides, hormonal peptides of the distal gut, biogenic amines, and other various mediators of the immune system [15, 26]. Although the factors that govern mucus discharge are partially defined for mammals, they are not well studied in fish. El-Salhy *et al*. [44] observed that the entero-endocrine L cells that produce peptide YY in the human colon, produce long, cytoplasmic processes connecting with neighbouring goblet cells thereby suggesting a paracrine signalling between cells resulting in mucus secretion. Lelievre *et al*. [45] suggested a pro-secretory role for the vasoactive intestinal peptide (VIP) in the goblet cells of the gut, as they found that the ratio of “full” goblet cells versus the number having secreted mucus was higher in C57BL/6 mice which lack the VIP gene. In common carp, *Cyprinus carpio* L., Neuhaus *et al*. [24] suggested that changes in mucin synthesis and secretion could be mediated primarily by local reactions in the gut and not by systemic immune mechanisms. The comments of the latter study also apply to the findings of the current study, however, the association of the various ECs with the secretion of specific fish regulatory substances requires further investigation.

The intimate relationship between the endocrine and mucous cells of the intestinal epithelium of both uninfected and *P. laevis*-infected *S. cephalus* has been demonstrated here by a combination of histochemical, immunohistochemical, immunofluorescence and TEM-based methodologies. This study represents the first time that a combined immunohistochemical method for endocrine cell markers and a histochemical method for the staining of gut glycoconjugates has been applied to investigate cellular differences in uninfected and helminth-infected piscine intestinal sections. The subsequent double immunofluorescence protocol using CLSM allowed for a clear characterisation of the endocrine and mucous cells and their relationship to one another. Using this approach, it has been possible to demonstrate the intimate contact between various IR ECs with mucous cells within the intestines of *S. cephalus;* the number of IR ECs was significantly higher in helminth-infected fish than in uninfected conspecifics. The methodologies employed here have allowed for the ECs with different morphologies to be catalogued, including the description of one type of EC which we refer to as “reservoir-like cells” in this account.

In the current study, TEM was also used to document the close contact and the presence of junctions between ECs and mucous cells from the mid-gut of *S. cephalus*. Similar connections between these two cell types have been described from the intestines of a number of teleosts including those reported by Noaillac-Depeyer & Gas [11], Rombout & Taverne-Thiele [33], and by Elbal *et al*. [34]. The ultrastructure of the ECs of *S. cephalus* bear some resemblance to those found in the stomach of European perch, *Perca fluviatilis* L., and the brown bullhead catfish, *Ameiurus nebulosus* [11] and in the gut of the leaping mullet, *Liza saliens* [34]. The ECs in the stomach of *P. fluviatilis* have well-developed Golgi complexes; the formation of secretory granules is discernible in the cisterna of the Golgi apparatus [11]. The Golgi apparatus in the ECs from the mid-gut of *S. cephalus,* however, was very scarce; secretory granules were seen in close proximity to well-developed rough endoplasmic reticulum (RER) and free ribosomes. There is a link between the extent of organelle development and its function in a cell. It is well known that, gut ECs principally secrete peptides and the function of the RER is intimately bound to the production and transport of proteins in the cell [46]. Thus, it is reasonable to suggest that the RER is very active in the formation of the secretory granules, and their contents, found in ECs.

There is no complete agreement on the role of excessive mucus secretion which, in the intestines of vertebrates infected with helminths, appears as an adherent blanket of mucus/gel. Although it has been suggested that increased mucus production in mammals may facilitate the expulsion of intestinal nematodes [47], this is not the case in the current *P. laevis-S. cephalus* situation. The barbed proboscis of *P. laevis* ensures a secure attachment to the intestinal wall of its host and no dislodged acanthocephalans were found at post-mortem. Thus we concur with the general suggestion that, the main role of mucus is to protect the underlying intestinal mucosa as a physical barrier against the mechanical and biochemical damage induced by parasites [48, 49].

## Conclusions

From the combined staining (*i.e.* histochemical, immunohistochemical, and immunofluorescence) and TEM-based methodologies applied here, the study has been able to demonstrate that: (a) intestinal infections of the acanthocephalan *P. laevis* result in increases in the number of mucous cells, most notably those containing acidic glycoconjugates and mixed glycoconjugates; (b) the presence of *P. laevis* induces an increase in the number of enteric endocrine cells that are immunoreactive to leu- and met-enkephalin, galanin, and serotonin anti-sera; (c) the enhanced mucus secretion in the helminth-infected intestines could be elicited by an increase in the number of endocrine cells which release various regulatory substances; and, (d) there is intimate contact between the intestinal mucous cells and endocrine cells of *S. cephalus*.

## Abbreviations

AB: Alcian blue
AB/PAS: Alcian blue / periodic acid Schiff
CLSM: Confocal laser scanning microscope
DBA: *Dolichos biflorus* agglutinin
DES: Diffuse endocrine systems
ECs: Endocrine cells
IR: Immunoreactive
PAS: Periodic acid Schiff
RER: Rough
RT: Room temperature
SE: Standard error
TBS: Tris buffered saline
TEM: Transmission electron microscopy
VIP: Vasoactive intestinal peptide
